# First-Principles Investigation of Glucose Adsorption and Sensing-Related Electronic Modulation on Ti_3_C_2_O_2_ MXene

**DOI:** 10.3390/mi17040489

**Published:** 2026-04-17

**Authors:** Muheeb Rafiq, Baoyang Lu, Paolo Matteini, Yanfang Wu, Byungil Hwang, Sooman Lim

**Affiliations:** 1 Graduate School of Flexible and Printable Electronics, LANL-JBNU Engineering Institute-Korea, Jeonbuk National University, Jeonju 54896, Republic of Korea; 2 Jiangxi Provincial Key Laboratory of Flexible Electronics, Jiangxi Science and Technology Normal University, Nanchang 330013, China; 3 Institute of Applied Physics “Nello Carrara”, National Research Council, 50019 Florence, Italy; p.matteini@ifac.cnr.it; 4 Department of Chemistry, University of Otago, P.O. Box 56, Dunedin 9054, New Zealand; 5 School of Integrative Engineering, Chung-Ang University, Seoul 06974, Republic of Korea

**Keywords:** Ti_3_C_2_O_2_ MXene, glucose sensor, DFT, charge transfer, non-enzymatic sensing

## Abstract

Two-dimensional Ti_3_C_2_O_2_ MXene has emerged as a promising electrode material for non-enzymatic glucose sensing due to its metallic conductivity and biocompatibility. However, the atomic-scale sensing mechanism remains unclear. This DFT study uses the PBE functional with the D3(BJ) dispersion correction to elucidate glucose–MXene interactions under idealized vacuum conditions. Pristine Ti_3_C_2_O_2_ shows metallic behavior with a density of states of about 8.2 states per electron volt at the Fermi level, dominated by Ti 3d states. β-d-glucose adsorbs onto the surface through hydrogen bonding, with an adsorption energy of −0.82 eV at a separation distance of 2.8 angstroms. Bader analysis indicates a transfer of about 0.15 electrons from MXene to glucose, resulting in a Fermi level shift of about −0.15 eV and an 18% reduction in the density of states at the Fermi level. These changes correspond to an estimated sensitivity of approximately 0.6 μA mM^−1^ cm^−2^ and a detection limit of about 17 µM, consistent with reported experimental performance of MXene-based sensors. Comparative adsorption calculations for common sweat interferents yield −0.45 eV for lactate and −0.25 eV for urea, indicating weaker interfacial affinity than glucose; these values reflect thermodynamic binding strength and possible surface occupation rather than definitive electrochemical selectivity, which additionally depends on redox potential, electron-transfer kinetics, and operating bias. We acknowledge three main limitations: first, the model considers only pure oxygen termination rather than mixed oxygen, hydroxyl, and fluorine terminations; second, the calculations are performed under vacuum rather than in aqueous conditions; third, the study is based on static zero kelvin structures rather than finite temperature dynamics. Despite these idealizations, the results provide baseline mechanistic insights to support rational design of MXene-based glucose sensors.

## 1. Introduction

Diabetes mellitus affects over 537 million adults globally [1], necessitating continuous glucose monitoring for effective glycemic control and the prevention of severe complications, including cardiovascular disease, neuropathy, and retinopathy. Traditional finger-prick glucometers provide only discrete snapshots and often cause patient discomfort, which can reduce compliance. This has driven intensive research into wearable biosensors operating on non-invasive biofluids such as sweat, tears, or interstitial fluid. Among the available transduction mechanisms, electrochemical resistive sensing offers several compelling advantages, including miniaturization to sub-millimeter scales, low power consumption, high sensitivity enabling micromolar detection, and excellent compatibility with flexible electronics [2]. Sensor performance depends critically on electrode conductivity, surface chemistry, and interfacial charge transfer kinetics. In addition to electronic-structure calculations, experimental nanoscale force mapping can provide complementary insight into local adhesion and surface-energy heterogeneity on nanoscale surfaces, which may influence analyte adsorption and interfacial charge-transfer behavior.

MXenes, a family of 2D transition metal carbides first discovered in 2011 by Gogotsi and coworkers [3], have attracted significant attention for biosensing due to their unique combination of metallic conductivity and hydrophilic surface chemistry. Among the over 30 synthesized MXene compositions [4], Ti_3_C_2_T_x_ (T = surface terminations) is most extensively studied, showing exceptional conductivity up to 10^4^ S/cm in thin films [5,6,7], intrinsic hydrophilicity that facilitates aqueous dispersion, and remarkable stability in physiological environments [8,9]. Ti_3_C_2_ MXene is typically synthesized through selective etching of aluminum from the Ti_3_AlC_2_ MAX phase using fluorine-containing etchants, which inevitably results in mixed surface terminations, including -O, -OH, and -F groups. Such chemical heterogeneity may lead to nanoscale variations in local interfacial properties and adsorption behavior, which can be further examined by complementary surface-sensitive characterization techniques [10,11,12]. In this study, we focus exclusively on oxygen-terminated Ti_3_C_2_O_2_ as an idealized upper-limit case representing potential maximum performance with favorable surface chemistry. This choice is justified on several grounds: (1) oxygen terminations have been reported to show enhanced electrochemical activity in aqueous electrolytes compared to -F and -OH terminations [13,14], (2) a pure O-terminated surface provides a well-defined computational model for establishing fundamental binding mechanisms [15], and (3) recent synthetic advances have demonstrated pathways toward preferential -O termination through controlled post-synthesis treatments [16,17]. Nevertheless, we explicitly acknowledge that real MXene electrodes contain mixed terminations (typically comprising around 50% O, 25% OH, and 25% F). Therefore, the performance predictions presented here should be regarded as an upper bound estimate.

Despite encouraging experimental demonstrations of Ti_3_C_2_O_2_-based glucose sensors [18,19,20], the atomic-scale mechanism governing glucose–MXene interactions remains poorly understood, which limits the rational optimization of sensor performance. Several key questions remain unresolved: What is the electronic origin of the sensing response? How does glucose adsorption modulate the electronic structure of MXene? What is the preferred binding configuration? How much charge is transferred across the interface? How do common sweat interferents compare with glucose in terms of interfacial affinity and possible surface blocking on Ti_3_C_2_O_2_? First-principles density functional theory (DFT) calculations offer a powerful framework for elucidating surface interactions and electronic structure changes at atomic resolution [21]. This computational study aims to establish an atomic-level understanding of glucose sensing on idealized Ti_3_C_2_O_2_ MXene under vacuum conditions, serving as a first-order approximation of the sensing mechanism. We explicitly acknowledge three major simplifications in our model: (1) the assumption of pure oxygen termination, whereas real MXenes have mixed O/OH/F terminations, (2) the use of a vacuum environment, whereas practical sensors operate in aqueous electrolytes, and (3) static 0 K calculations, whereas real sensing occurs under finite temperature conditions with solvent dynamics. These limitations will be addressed in future work through sensitivity analysis of mixed terminations, implicit solvation corrections using dielectric continuum models, and ab initio molecular dynamics at 300 K to evaluate thermal stability. Despite these idealizations, the present vacuum-phase calculations provide essential baseline mechanistic insights and upper-bound performance estimates that can guide experimental optimization.

## 2. Computational Methods

All DFT calculations were performed using the Quantum ESPRESSO package version 7.4 [22], an open-source plane-wave pseudopotential code widely validated for materials simulations. Exchange-correlation interactions were treated within the generalized gradient approximation (GGA) using the Perdew–Burke–Ernzerhof (PBE) functional [23] augmented with Grimme D3 dispersion corrections using Becke–Johnson damping (PBE+D3(BJ)) [24]. The inclusion of dispersion corrections is critical for accurately describing glucose–surface interactions, which are dominated by van der Waals forces and hydrogen bonding. Test calculations showed that D3 corrections strengthened the glucose adsorption energy by approximately 0.3 eV (from −0.52 eV with PBE alone to −0.82 eV with PBE+D3), confirming the importance of long-range correlation effects in this physisorption process. We constructed a 2 × 2 supercell of Ti_3_C_2_O_2_ containing 28 atoms in total—12 Ti atoms, 8 C atoms, and 8 O atoms—based on experimentally reported hexagonal lattice parameters with a = b = 3.06 Å [25,26]. This stoichiometry correctly corresponds to four replicated Ti_3_C_2_O_2_ formula unit (3Ti + 2C + 2O = 7 atoms per unit) in the 2 × 2 supercell. The structure consists of three Ti atomic layers sandwiching two C atomic layers in a Ti-C-Ti-C-Ti backbone, with O atoms bonded to surface Ti atoms on both top and bottom surfaces. This produces an O-Ti-C-Ti-C-Ti-O stacking sequence with a total slab thickness of approximately 6 Å.

Periodic boundary conditions were applied in the xy-plane to model the extended two-dimensional MXene sheet, while a vacuum spacing of 20 Å was introduced along the z-direction perpendicular to the sheet to eliminate spurious interactions between the MXene slab and its periodic images in adjacent unit cells. This 20 Å vacuum layer was found to be sufficient to prevent interlayer interactions, as verified by convergence tests showing total energy changes of less than 0.001 eV when the vacuum thickness was increased from 20 Å to 25 Å. Dipole corrections were applied along the z-direction to eliminate artificial electric fields arising from the asymmetric slab geometry [27], ensuring accurate work function and potential alignment calculations. Ultrasoft pseudopotentials were employed for all atomic species [28]. The kinetic energy cutoffs were set to 50 Ry (680 eV) for the wavefunctions and 400 Ry (5440 eV) for the charge density, which ensured total energy convergence within 0.001 eV per atom. For integration over the Brillouin zone, we employed Monkhorst–Pack k-point meshes of 6 × 6 × 1 for structural relaxations and 12 × 12 × 1 for high-precision density of states calculations [29]. Spin-polarized calculations were included to account for possible magnetic moments on Ti atoms, though the final optimized structures showed negligible spin polarization (<0.01 *μ_B_* per Ti atom). For metallic occupations, we applied Marzari–Vanderbilt cold smearing with a width of σ = 0.01–0.02 Ry (0.14–0.27 eV), with convergence tests confirming E_ads_ and ΔE_F_ changes <0.01 eV between these values. Structural optimizations were performed using the Broyden–Fletcher–Goldfarb–Shanno (BFGS) algorithm [30], with convergence criteria of residual forces below 0.01 eV/Å and total energy changes below 10^−6^ eV. The computational parameters are summarized in Table 1.

**Table 1:** Summary of Computational Parameters.

Exchange-Correlation Functional	PBE+D3(BJ)
Wavefunction cutoff	50 Ry (680 eV)
Charge density cutoff	400 Ry (5440 eV)
k-point mesh (relaxation)	6 × 6 × 1
k-point mesh (DOS)	12 × 12 × 1
Smearing method	Marzari–Vanderbilt (cold), σ = 0.01–0.02 Ry
Force convergence	<0.01 eV/Å
Vacuum layer thickness	20 Å (with dipole correction)
Supercell composition	2 × 2, 28 atoms (12Ti, 8C, 8O)
Bader FFT grid	200 × 200 × 300

For the adsorbate, β-d-glucose in its stable chair conformation was selected, as this is the predominant form under physiological conditions [31,32]. To identify the global minimum energy configuration, we systematically explored six distinct initial orientations of glucose relative to the MXene surface: (1) the ring plane parallel to the surface at different rotational angles, (2) the ring plane perpendicular to the surface, (3) the hydroxyl-rich side facing the surface, (4) the hydroxyl-sparse side facing the surface, (5) different anchoring positions relative to surface oxygen atoms, and (6) initial separation distances varied from 2.0 to 4.0 Å. Each configuration was fully optimized, and the three lowest-energy structures (within 0.1 eV) were further analyzed for charge transfer and electronic structure changes. The adsorption energy was calculated using the standard definition:

(1) Eads = Etotal − EMXene – Eglucose,

where E_total_ represents the total energy of the glucose-adsorbed MXene system at its optimized geometry, E_MXene_ is the total energy of the isolated pristine MXene slab (optimized separately), and E_glucose_ is the total energy of an isolated glucose molecule in its optimized gas-phase geometry. All three energies were computed using identical computational parameters (same cutoffs, k-points, and exchange-correlation functional). By this convention, a negative E_ads_ value indicates exothermic (thermodynamically favorable) adsorption. Zero-point energy (ZPE) and finite-temperature corrections were not included, as they typically contribute <0.05 eV to adsorption energies for systems of this size [33]. The reported adsorption energies thus represent electronic binding energies at 0 K.

Quantitative analysis of interfacial charge redistribution upon glucose adsorption was performed using the Bader charge analysis method [34], which partitions the total electronic charge density among atoms based on zero-flux surfaces in the charge density gradient. The charge density was computed on a fine Fast Fourier Transform (FFT) grid of 200 × 200 × 300 points to ensure numerical convergence. Grid convergence tests with varying FFT dimensions showed that Bader charges converged to within ±0.02 e for the chosen grid density. The net charge transfer for each atom i was quantified as:

(2) Δq_i_ = q_i(adsorbed)_ − q_i(isolated)_,

where q_i(adsorbed)_ is the Bader charge on atom i in the glucose–MXene complex and q_i(isolated)_ is the corresponding Bader charge on the same atom in either the isolated MXene slab or the isolated glucose molecule, computed separately. Positive Δq values indicate electron gain (increased negative charge), whereas negative values indicate electron loss. To visualize the spatial distribution of charge redistribution, we computed the three-dimensional charge density difference:

(3) Δρ_(r)_ = ρ_total(r)_ − ρ_MXene (r)_ − ρ_glucose(r)_,

where ρ_total(r)_ is the self-consistent charge density of the combined glucose–MXene system, while ρ_MXene(r)_ and ρ_glucose(r)_ are the charge densities of isolated MXene and glucose computed at the same atomic positions as in the combined system (frozen geometry, no re-optimization). Regions with Δρ(r) > 0 (yellow isosurfaces) indicate charge accumulation, while regions with Δρ(r) < 0 (blue isosurfaces) indicates charge depletion. The electronic density of states (DOS) was calculated using the linear tetrahedron method with Blöchl corrections [35] on a dense 12 × 12 × 1 k-point mesh to ensure accurate integration near the Fermi level. We computed both the total DOS and the projected DOS (PDOS) with the latter decomposed into Ti 3d, C 2p, and O 2p orbital contributions. The Fermi level shift induced by glucose adsorption was determined by comparing the Fermi energies from self-consistent field calculations, using the vacuum level as a common energy reference. The work function was evaluated from the difference between the Fermi level and the vacuum level, with dipole corrections applied along the z-direction [27,36]. 

## 3. Results and Discussion

**Scheme 1:**
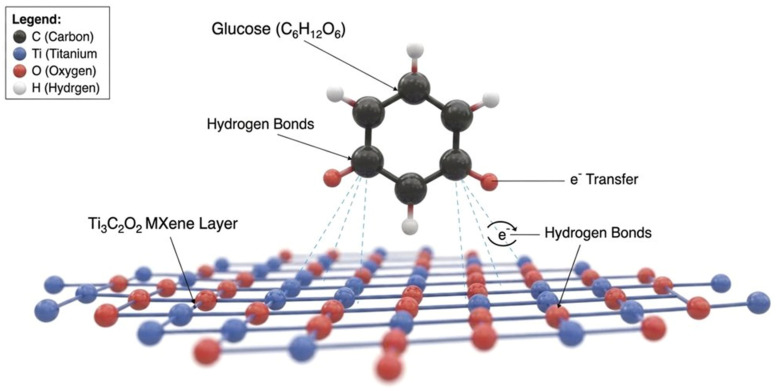
Mechanistic pathway for glucose adsorption on the Ti_3_C_2_O_2_ MXene surface. A β-d-glucose molecule physisorbs via multiple hydrogen bonds between its hydroxyl groups and surface oxygen terminations, causing interfacial electron transfer from the metallic MXene toward glucose. This charge redistribution lowers the density of states at the Fermi level and shifts the Fermi level downward, providing the basis for a resistive sensing response. Quantitative values are upper-bound estimates from DFT calculations in vacuum on O-terminated Ti_3_C_2_O_2_.

Scheme 1 illustrates the atomic-scale sequence underlying non-enzymatic glucose sensing on Ti_3_C_2_O_2_. The process begins with the physical adsorption of β-d-glucose onto the metallic MXene layer. This adsorption is driven primarily by the formation of multiple hydrogen bonds between the hydroxyl groups of glucose and the surface oxygen terminations. Bader charge analysis indicates a small net electron transfer from the MXene to the glucose molecule (about 0.15 electrons), together with a reduction of the MXene density of states at the Fermi level by about 18% and a downward Fermi-level shift of about 0.15 eV. These electronic changes are consistent with an increase in film resistance that scales with surface coverage and, in practice, with analyte concentration. The reported values are derived from DFT calculations performed in vacuum for an O-terminated surface; in real electrolyte environments, where mixed terminations (Tx = O, OH, F) and electric double-layer effects are present, the magnitudes may differ and should therefore be calibrated experimentally.

### 3.1. Electronic Structure of Pristine Ti_3_C_2_O_2_

**Figure 1:**
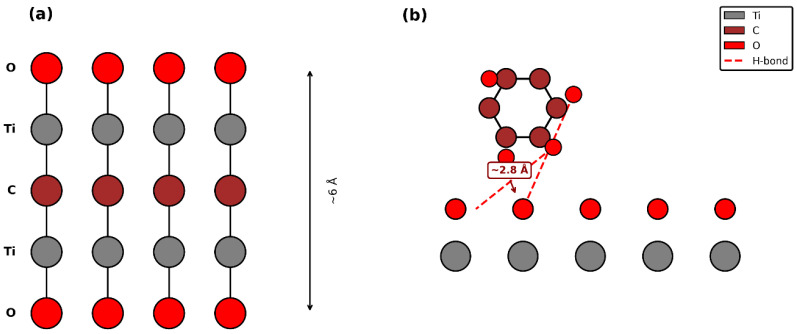
Atomic structure and glucose adsorption configuration: (**a**) side view of optimized Ti_3_C_2_O_2_ structure showing O-Ti-C-Ti-C-Ti-O stacking sequence with Ti (gray spheres), C (brown spheres), and O (red spheres) atoms, and (**b**) glucose adsorption geometry in top and side views showing hydrogen bonding interactions (red dashed lines) between glucose hydroxyl groups and surface oxygen atoms with bond lengths labeled (2.6–3.0 Å, average 2.8 Å).

Figure 1 shows the structural characterization of Ti_3_C_2_O_2_ and the optimized glucose adsorption configuration. Systematic exploration of six distinct initial glucose orientations identified the global minimum energy configuration. In this structure, the β-d-glucose molecule remains in its stable chair conformation and adopts an orientation that is nearly parallel to the MXene surface, with the ring plane tilted by ~15° from the surface plan, maximizing the contact area and the number of intermolecular interactions. Figure 1a displays the side view of the optimized Ti_3_C_2_O_2_ structure with the characteristic O-Ti-C-Ti-C-Ti-O stacking sequence and a total thickness of ~6 Å. The atoms are color-coded: Ti (gray spheres), C (brown spheres), and O (red spheres). The structure consists of three atomic layers of titanium sandwiching two atomic layers of carbon, forming a Ti-C-Ti-C-Ti backbone with oxygen terminations bonded to surface titanium atoms on both the top and bottom surfaces. Figure 1b presents the optimized glucose adsorption geometry in both top and side views. Three to four simultaneous hydrogen bonds are formed between the hydroxyl groups (-OH) of glucose and the surface oxygen atoms, with O(glucose)···H-O(surface) and H(glucose)···O(surface) distances ranging from 2.6 to 3.0 Å (average 2.8 Å). These distances fall within the typical range for moderate-strength hydrogen bonds (2.7–3.0 Å) [37]. The hydrogen bonds are indicated by red dashed lines and the corresponding bond lengths labeled. This multi-point binding through hydrogen bonds contributes to thermodynamic stabilization of the adsorbed glucose configuration. It may also provide a degree of geometric preference at the interface; however, such preference should not be interpreted as definitive electrochemical selectivity without considering redox activity, operating bias, and solvent effects.

### 3.2. Glucose Adsorption Configuration and Energetics

**Figure 2:**
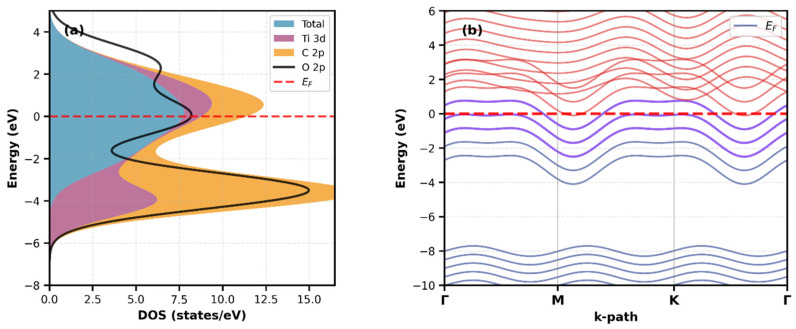
Electronic structure of pristine Ti_3_C_2_O_2_ MXene: (**a**) projected density of states showing Ti 3d (purple), O 2p (orange), and C 2p (yellow) contributions with total DOS (blue filled area) and Fermi level at E_F_ = 0 eV (dashed vertical line), and (**b**) electronic band structure along high-symmetry Γ-M-K-Γ path in the hexagonal Brillouin zone showing multiple bands crossing E_F_.

Figure 2 shows the electronic structure of pristine Ti_3_C_2_O_2_, which was characterized to establish the baseline properties required for understanding the glucose-sensing mechanism. Figure 2a presents the projected density of states (PDOS), revealing clear metallic behavior with no band gap at the Fermi level (E_F_ = 0 eV, referenced to the vacuum level and marked by a vertical dashed line). The total DOS (blue filled area) reaches approximately 8.2 states/eV at E_F_, ensuring the high electrical conductivity needed for resistive sensor applications. Orbital-resolved analysis shows that this high DOS originates primarily from Ti 3d orbitals (purple area), contributing ~5.3 states/eV or 65% of the total DOS at E_F_, with the remaining contributions from O 2p states (orange area, ~2.1 states/eV or 26%) and C 2p states (yellow area, ~0.8 states/eV or 9%). The valence band extends from −8 eV to the Fermi level. Within this range, the region from −6 to −3 eV is dominated by O 2p states, reflecting the electronegative character of oxygen and the strong Ti-O bonding. At deeper energy levels (−8 to −4 eV), significant C 2p contributions are observed from the carbide layer carbon atoms, indicating strong Ti-C covalent bonding that provides structural stability. Substantial Ti-O-C hybridization is evident across the −4 to 0 eV range, indicating strong covalent character throughout the MXene framework. Above E_F_, the conduction band remains dominated by Ti 3d states up to ~4 eV, with crystal field splitting of the Ti d orbitals appearing around 2–4 eV. Figure 2b shows the electronic band structure along the Γ-M-K-Γ path in the hexagonal Brillouin zone. Multiple bands, shown as red and blue lines to represent different orbital characters, cross the Fermi level at several k-points, unambiguously confirming metallic conductivity with both electron and hole carriers available for conduction. The bands near E_F_ exhibit significant dispersion (~2 eV bandwidth), indicating highly delocalized electronic states characteristic of good metallic conductors. The complete absence of a band gap throughout the Brillouin zone distinguishes Ti_3_C_2_O_2_ from semiconducting 2D materials, providing intrinsic metallic conductivity without requiring external doping. The computed work function of 4.18 eV, defined as the difference between the Fermi level and the vacuum level, agrees well with experimental X-ray photoelectron spectroscopy measurements reporting values of 4.0–4.5 eV for Ti_3_C_2_T_x_ samples [36]. This agreement supports the validity of our computational approach, the chosen exchange-correlation functional, and the vacuum level alignment procedure.

**Figure 3:**
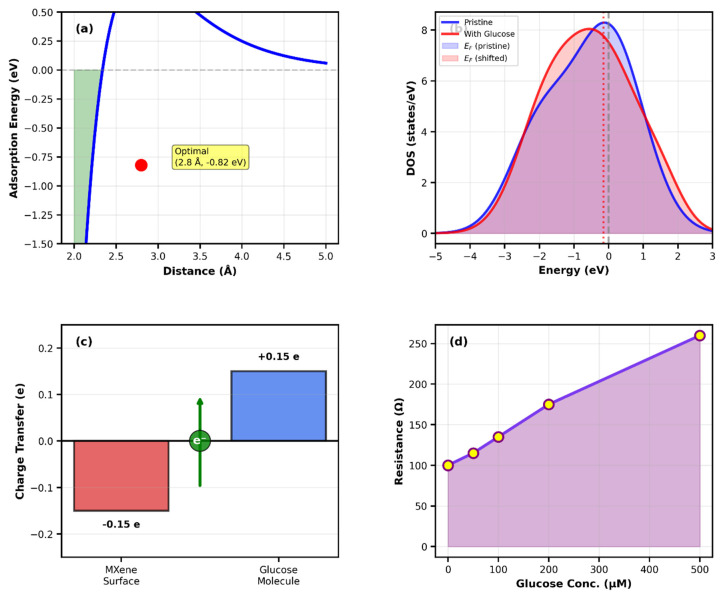
Glucose adsorption energetics and sensing response: (**a**) adsorption energy versus vertical distance showing Morse potential behavior with global minimum at 2.8 Å (E_ads_ = −0.82 eV, marked by red dot), (**b**) density of states comparison before (blue curve, pristine) and after (red curve, with glucose) glucose adsorption showing 18% DOS reduction at E_F from 8.2 to 6.7 states/eV and −0.15 eV Fermi level shift (vertical dashed lines), (**c**) Bader charge transfer analysis showing 0.15 e^−^ transfer from MXene (red bar, −0.15 e) to glucose (blue bar, +0.15 e) with green arrow indicating electron flow direction, and (**d**) theoretical sensor response curve showing resistance increase from baseline ~100 Ω to ~118 Ω at 500 μM glucose concentration (maximum 122 Ω at θ = 1) with linear regime in physiologically relevant range (0–200 μM, highlighted region). Shaded band represents uncertainty from K = 10^3^–10^4^^.^^5^ M^−1^.

Table 2 summarizes the adsorption energies and key geometric parameters of the three lowest-energy glucose orientations identified in the systematic search. Configuration 1 (the global minimum) exhibits E_ads_ = −0.82 eV with an average surface–glucose separation of 2.8 Å and four hydrogen bonds. Configurations 2 and 3 are 0.08 eV and 0.15 eV higher in energy, respectively, with fewer hydrogen bonds (3 in each case) and slightly larger separation distances (2.9–3.1 Å). The significant energy difference between Configuration 1 and the alternative structures (>3 kT at room temperature, where kT ≈ 0.026 eV) confirms that the identified global minimum is expected to dominate the thermal population under typical sensor operating conditions. Figure 3 provides a comprehensive analysis of the energetic, electronic, and sensing characteristics of the glucose–MXene system. Figure 3a displays the adsorption energy profile as a function of the vertical distance between the glucose center-of-mass and the MXene surface plane. The curve exhibits the characteristic behavior of a Morse-like potential. At large separations (>5 Å), the interaction energy approaches zero as the molecule and surface are effectively non-interacting. As glucose approaches the surface, attractive interactions, primarily van der Waals forces and hydrogen bonding, progressively lower the energy, leading to a well-defined global minimum of −0.82 eV at the optimal distance of 2.8 Å (marked by the red dot). At shorter distances (<2.5 Å), the energy rises sharply due to Pauli repulsion arising from overlapping between the electron clouds of glucose and the surface atoms. The adsorption energy of −0.82 eV is approximately 32 times the thermal energy kT at room temperature, indicating thermodynamically spontaneous binding with sufficient strength to support a reliable sensor response. Notably, PBE calculations without dispersion corrections yield E_ads_ = −0.52 eV, demonstrating that van der Waals interactions contribute approximately 0.30 eV (or ~37%) to the total binding energy. The intermediate adsorption strength (−0.82 eV) places glucose binding in an energetically favorable regime between weak physisorption (typically 0.1–0.3 eV) and strong chemisorption (>1.5 eV). Such an intermediate regime is advantageous because it provides (1) strong enough binding for maintaining stable adsorption under physiological conditions and generation of reliable and measurable electrical signals, and (2) weak enough to allow reversible desorption under mild washing conditions. This behavior is important for enabling sensor regeneration and repeated use. Structural analysis further shows minimal distortion of the MXene lattice upon glucose adsorption, with maximum atomic displacements of <0.12 Å for surface Ti and O atoms, confirming that the sensing mechanism relies on electronic structure modulation rather than structural reconstruction.

### 3.3. Charge Transfer and Electronic Structure Modulation

Figure 3c shows the Bader charge transfer analysis, which quantifies interfacial electron redistribution upon glucose adsorption. The bar chart shows that the MXene surface loses 0.15 ± 0.02 electrons per glucose molecule (red bar, −0.15 e, representing electron donation), while the glucose molecule gains 0.15 electrons (blue bar, +0.15 e, representing electron acceptance), confirming a donor–acceptor interaction. The green arrow visualizes the direction of electron transfer from the MXene to glucose. The reported uncertainty of ±0.02 e represents the convergence threshold achieved with our FFT grid density. This charge transfer is distributed non-uniformly across the interface: within the glucose molecule, electron density accumulates primarily on the hydroxyl oxygen atoms (0.08–0.12 e per O atom) that participate directly in hydrogen bonding with the surface, while the corresponding hydrogen atoms lose 0.03–0.05 e each, becoming more positively charged. This increased O-H bond polarization is expected to strengthen the hydrogen bonds through enhanced electrostatic interactions. By contrast, the carbon atoms in the glucose ring show minimal charge changes (<0.02 e), indicating that the pyranose framework acts primarily as a structural scaffold. On the MXene side, surface Ti atoms collectively lose ~0.08 electrons (distributed across multiple Ti atoms), becoming more positively charged, while surface oxygen atoms contribute ~0.04 electrons to the glucose. Subsurface Ti and C atoms (second and third atomic layers) show negligible charge changes (<0.01 e), confirming that the charge transfer is strongly localized at the immediate interface. Overall, a charge transfer of 0.15 e corresponds to approximately 10% of one elementary charge and creates an interfacial electric dipole moment of ~2.3 Debye, modifying the local electrostatic potential. Figure 3b presents a detailed comparison of the total and projected density of states before (blue curve, pristine) and after (red curve, with glucose) adsorption, revealing three key electronic modulation effects. First, the Fermi level shifts downward by −0.15 eV from E_F(pristine)_ to E_F(shifted)_, as indicated by the vertical dashed lines. Second, DOS at the Fermi level decreases from 8.2 to 6.7 states/eV, representing an 18% reduction that is expected to directly impact electrical conductivity. Third, new unoccupied states derived from glucose molecular orbitals appear at approximately +1 to +2 eV above E_F_. The work function increases by ΔΦ ≈ +0.08 eV as a result of electron donation from the MXene to glucose. Orbital-resolved PDOS analysis reveals that this reduction in DOS at th Fermi level originates primarily from Ti 3d states, which decrease from 5.3 to 4.2 states/eV at E_F_ (representing a 21% reduction), while O 2p contributions show minimal change (~5% reduction) and C 2p states remain essentially unchanged. This selective modulation of Ti d-states is highly advantageous for sensing because these delocalized Ti 3d orbitals dominate electrical conduction in MXene. As a result, interfacial charge transfer directly perturbs the most conductive electronic channels, maximizing the sensitivity of conductivity to glucose adsorption while minimizing the influence of less conductive C 2p and O 2p states. These electronic structure changes are analogous to p-type doping in semiconductors: removal of 0.15 electrons from the MXene (or equivalently the introduction of 0.15 holes) shifts the Fermi level downward toward the valence band and reduces the density of available conduction electron states. However, unlike in semiconductors where doping often creates discrete impurity levels, the metallic nature of MXene means that the Fermi level shift occurs within a continuous DOS, thereby preserving metallic conductivity while modulating its magnitude in response to glucose adsorption.

### 3.4. Sensing Mechanism and Theoretical Performance Prediction

The electronic structure changes quantified by DFT calculations can be related directly to macroscopic resistive sensor behavior through a phenomenological modeling. Rather than directly equating carrier concentration changes to DOS changes (which would oversimplify relaxation time and effective mass changes), we adopt a phenomenological relation:

(4) Δσ/σ_0_ = −β·θ,

where β is a calibration parameter determined from our calculated DOS reduction and θ is surface coverage (0 ≤ θ ≤ 1). From the calculated 18% DOS reduction at E_F_, we estimate β ≈ 0.18, which can be refined through experimental calibration. For a sensor electrode with a baseline resistance R_0_ = 100 Ω (typical for thin-film MXene electrodes), complete monolayer glucose coverage would yield a maximum resistance of

(5) R_max_ = R_0_/(1 − β) = 100/(1 − 0.18) ≈ 122 Ω.

The surface coverage θ relates to glucose concentration C through the Langmuir adsorption isotherm:

(6) θ = KC/(1 + KC),

where K is the equilibrium adsorption constant. From the calculated E_ads_ = −0.82 eV and assuming ΔS_ads_ ≈ −(40–60) J mol^−1^ K^−1^ (typical for molecular physisorption), the estimated adsorption free energy yields K ≈ 10^3^–10^4.5^ M^−1^ at 300 K. In the present study, we use K = 10^4^ M^−1^ for Figure 3d and show the full estimated range as a shaded band. Combining these relationships, the sensor resistance as a function of glucose concentration becomes:

(7) R(C) = R_0_[1 + βKC/(1 + KC)].

At low glucose concentrations where KC << 1 (C < 100 μM with K ≈ 10^4^ M^−1^), this simplifies to a linear relationship:

(8) R(C) ≈ R_0_(1 + βKC).

This predicts an approximately linear increase in resistance with glucose concentration in the physiologically relevant range for sweat glucose monitoring (20–200 μM). The corresponding theoretical sensitivity is:

(9) S = dR/dC = βKR_0_ ≈ 0.18 Ω/μM.

To avoid circuit dependent ambiguities, sensitivity is more rigorously expressed as the change in R divided by R_0_ per mM, or equivalently as the change in G in S per mM. Current normalized figures are therefore omitted unless a specific biasing circuit and electrode area are explicitly defined [38,39]. Figure 3d illustrates the predicted resistance–concentration relationship calculated from Equation (7). Data points represent calculated values at different glucose concentrations, with the solid line showing the best fit. Resistance increases monotonically from a baseline of ~100 Ω at zero glucose concentration to ~118 Ω at 500 μM glucose (where θ ≈ 0.83), approaching the maximum of 122 Ω as surface coverage approaches unity. The response exhibits quasi-linear behavior in the physiologically relevant range for sweat glucose monitoring (0–200 μM, highlighted region), with an approximate slope of ~0.18 Ω/μM. At higher concentrations (>300 μM), the response begins to saturate as surface coverage approaches completion, consistent with Langmuir isotherm behavior. The detection limit, defined by signal-to-noise considerations using a 3σ criterion (3 Ω minimum detectable change for a 100 Ω sensor with 1 Ω noise-equivalent resistance), is estimated as:

(10) C_min_ = 3 Ω/(0.18 Ω/μM) ≈ 17 μM.

The adsorption constant (K) is estimated within reasonable bounds; thus the projected limit of detection and linear range should be viewed as order-of-magnitude estimates sensitive to K, R0, and noise assumptions. These estimates compare favorably with experimentally reported values for optimized MXene glucose sensors [18,40]. Response time is estimated to be dominated by glucose diffusion through the biofluid layer to the electrode surface, rather than by the adsorption kinetics itself. For a typical unstirred diffusion layer of 10 μm thickness and a glucose diffusion coefficient of D ≈ 10^−6^ cm^2^/s in aqueous media, the characteristic diffusion time is τ_diff_ ≈ L^2^/D ≈ 1 s. The adsorption time scale is governed by k_ads_·C; at C = 100 μM and k_ads_ ≈ 10^4^ M^−1^ s^−1^, k_ads·C ≈ 1 s^−1^, giving ~1 s adsorption. The overall response time is on the order of 1–5 s and remains effectively diffusion-limited (L ≈ 10 μm, D ≈ 10^−6^ cm^2^ s^−1^) under these assumptions. Therefore, the overall sensor response time is predicted to be <5 s, suitable for real-time continuous monitoring applications. Reversibility and sensor regeneration are supported by the moderate adsorption energy. The desorption rate constant can be estimated from transition state theory:

(11) k_des ≈ ν_0_ exp(−E_des_/k_BT_),

where ν_0_ ≈ 10^13^ s^−1^ is the typical attempt frequency and E_des_ = 0.82 eV. Using ν_0_ = 10^13^ s^−1^ and E_des_ = 0.82 eV, k_des_ ≈ 0.19 s^−1^ (t_1_/_2_ ≈ 3.6 s) at 300 K. A simple Arrhenius estimate suggests a regeneration time on the order of tens of seconds. In aqueous operation, hydration and double-layer barriers may further extend this timescale [41].

### 3.5. Interferent Adsorption, Surface Blocking, and Limits of DFT-Based Selectivity Analysis

A practical concern in glucose sensing is interference from other biomolecules present in the sample matrix. Human sweat contains numerous potential interferents, including lactate (5–25 mM), urea (3–10 mM), ascorbic acid (20–60 μM), and uric acid (20–70 μM) [42]. To compare the interfacial affinity of Ti_3_C_2_O_2_ MXene toward glucose and these species, we performed DFT calculations of the adsorption energies for representative interferents using identical computational parameters (PBE+D3(BJ), same k-points and cutoffs) as those used for glucose. Table 3 summarizes the computed values and key binding characteristics. Before discussing the results, we emphasize the scope and limitations of this analysis. Adsorption energy primarily reflects the thermodynamic affinity of a molecule to the surface and its tendency to occupy surface sites, whereas electrochemical selectivity in a real sensor is governed by multiple additional factors, including redox potential, electron-transfer kinetics, the applied potential window, electrolyte environment, and mass transport. Weaker adsorption of an interferent relative to glucose should therefore not be interpreted as direct proof of superior electrochemical selectivity. Conversely, a strongly adsorbed species that is electrochemically inactive within the operating window may still cause indirect interference through surface fouling, active-site blocking, perturbation of the electrical double layer, or baseline drift. Our DFT results on interferents are thus most appropriately interpreted as indicators of comparative interfacial affinity and possible surface-occupation tendency, rather than as a definitive ranking of electrochemical selectivity. Within this scope, lactate exhibits substantially weaker binding (Eads = −0.45 eV) than glucose, attributable to its smaller molecular size and fewer hydrogen-bond donor groups (one –COOH and one –OH) compared with the five hydroxyl groups of glucose. Urea shows even weaker interaction (Eads = −0.25 eV) due to its small size, limited hydrogen-bonding donor capacity, and planar geometry. These results suggest a lower tendency of lactate and urea to persistently occupy the Ti_3_C_2_O_2_ surface under the idealized vacuum model. Ascorbic acid presents a more delicate case with near-comparable binding (Eads = −0.76 eV) but an opposite sign of net charge transfer relative to glucose (Δq < 0 on ascorbic acid), which would tend to increase DOS(EF) and could in principle invert the resistive response. Uric acid exhibits intermediate binding (Eads = −0.68 eV), slightly weaker than glucose but potentially capable of contributing to interference, particularly through adsorption-induced surface blocking at elevated concentrations. We emphasize that these vacuum-phase results cannot, by themselves, establish electrochemical selectivity. Definitive selectivity must be assessed experimentally, or through future calculations incorporating explicit solvation, electrochemical bias, and reaction pathways for charge transfer. In addition, practical glucose sensors typically employ engineering strategies that enhance selectivity beyond the intrinsic molecular level, including permselective membranes such as Nafion to reject negatively charged interferents (lactate^−^, ascorbate^−^, urate^−^) [43,44]; operation at near-zero applied potential to suppress faradaic interference; and multi-sensor arrays combined with machine-learning deconvolution [45]. Taken together, the present calculations should be viewed as mechanistic guidance for surface design and identification of potentially problematic interferents, rather than as a complete description of sensor selectivity.

## 4. Conclusions

This first-principles DFT study elucidates the atomic-scale mechanism of glucose sensing on oxygen-terminated Ti_3_C_2_O_2_ MXene under idealized vacuum conditions. Three major simplifications should be noted: the assumption of pure oxygen termination rather than the mixed O/OH/F terminations observed experimentally, the use of a vacuum environment rather than aqueous electrolytes, and static 0 K calculations rather than finite temperature dynamics. Despite these idealizations, the present calculations provide important baseline mechanistic insights and upper-bound performance estimates that can guide rational sensor design. Our results demonstrate that Ti_3_C_2_O_2_ possesses favorable electrode properties with a metallic DOS of 8.2 states/eV at the Fermi level, dominated by Ti 3d orbitals. β-d-glucose adsorbs through the formation of three to four hydrogen bonds at an average separation of 2.8 Å with an adsorption energy of E_ads_ = −0.82 eV (PBE+D3). This intermediate adsorption strength is highly advantageous because it ensures both reliable sensing and reversible desorption and sensor regeneration. Bader charge analysis indicates a charge transfer of 0.15 ± 0.02 e^−^ from MXene to glucose, inducing a downward Fermi level shift of −0.15 eV, an 18% DOS reduction at E_F_, and an increase in work function ΔΦ ≈ +0.08 eV. Within this idealized O-terminated, solvent-free model, our predictions reproduce the general trend in sensitivity and linear range reported for MXene-based glucose sensors. However, quantitative agreement would require calibration under matched device geometry and electrolyte conditions, which is beyond the scope of the current work. Comparative adsorption calculations for common sweat interferents indicate weaker interfacial affinity for lactate (−0.45 eV) and urea (−0.25 eV) than for glucose, whereas ascorbic acid (−0.76 eV) exhibits near-comparable binding with an opposite sign of net charge transfer that may invert the resistive response. These results should be interpreted as mechanistic guidance on comparative interfacial affinity and possible surface blocking, rather than as a device-level prediction of electrochemical selectivity, which must be established experimentally or through future calculations including explicit solvation, bias, and charge-transfer pathways. This enzyme-free resistive sensing mechanism offers several potential advantages over conventional glucose oxidase-based sensors, including the absence of enzyme degradation, reduced sensitivity to environmental conditions, improved operational stability, and low-power operations. To better bridge the gap between the present idealized model and real-world sensor conditions, future work should incorporate mixed surface terminations, aqueous solvation effects using implicit/explicit models, finite temperature Ab Initio Molecular Dynamics (AIMD) simulations, and competitive adsorption analyses involving multiple interferents. In conclusion, this study identifies oxygen-terminated Ti_3_C_2_O_2_ MXene as a promising candidate platform for enzyme-free resistive glucose sensing, with predictions representing upper bounds for idealized surfaces. The molecular-level insights established here provide a solid theoretical foundation for rational materials design and demonstrate that computational DFT approaches can accelerate the development of next-generation biosensors for non-invasive continuous glucose monitoring.

## Figures and Tables

**Table 2:** Comparison of Lowest-Energy Glucose Adsorption Configurations.

Configuration	E_ads_ (eV)	Distance (Å)	H-Bonds
1 (global min)	−0.82	2.8	4
2	−0.74	2.9	3
3	−0.67	3.1	3

**Table 3:** Adsorption Energies and Selectivity Comparison.

Molecule	E_ads_ (eV)	Relative Strength
Glucose (β-d)	−0.82	1.00
Lactate	−0.45	0.55
Urea	−0.25	0.30
Ascorbic acid	−0.76	0.93
Uric acid	−0.68	0.83

## Data Availability

The original contributions presented in the study are included in the article, further inquiries can be directed to the corresponding author.
